# Pars interarticularis screws for posterior cervical fusion – investigating a new trajectory using a CT-based multiplanar reconstruction: Part I

**DOI:** 10.1007/s00701-024-06184-x

**Published:** 2024-07-11

**Authors:** Sara Lener, Christoph Wipplinger, Anto Abramovic, Heiko Koller, Claudius Thomé, Michael Verius, Sebastian Hartmann

**Affiliations:** 1 Department of Neurosurgery, Medial University of Innsbruck, Anichstrasse 35, 6020 Innsbruck, Austria; 2 Department of Orthopedics and Traumatology, Paracelsus Medical University, Salzburg, Austria; 3 Department of Radiology, Medical University of Innsbruck, Innsbruck, Austria

**Keywords:** Pars interarticularis, Isthmus, Dorsal cervical stabilization, Dorsal cervical fusion, Novel trajectory

## Abstract

**Background:**

Lateral mass screw fixation is the standard for posterior cervical fusion between C3 and C6. Traditional trajectories stabilize but carry risks, including nerve root and vertebral artery injuries. Minimally invasive spine surgery (MISS) is gaining popularity, but trajectories present anatomical challenges.

Research Question.

This study proposes a novel pars interarticularis screw trajectory to address these issues and enhance in-line instrumentation with cervical pedicle screws.

**Materials and Methods:**

A retrospective analysis of reformatted cervical CT scans included 10 patients. Measurements of the pars interarticularis morphology were performed on 80 segments (C3-C6). Two pars interarticularis screw trajectories were evaluated: Trajectory A (upper outer quadrant entry, horizontal trajectory) and Trajectory B (lower outer quadrant entry, cranially pointed trajectory). These were compared to standard lateral mass and cervical pedicle screw trajectories, assessing screw lengths, angles, and potential risks to the spinal canal and transverse foramen.

**Results:**

Trajectory B showed significantly longer pars lengths (15.69 ± 0.65 mm) compared to Trajectory A (12.51 ± 0.24 mm; p < 0.01). Lateral mass screw lengths were comparable to pars interarticularis screw lengths using Trajectory B. Both trajectories provided safe angular ranges, minimizing the risk to delicate structures.

**Discussion:**

and Conclusion.

Pars interarticularis screws offer a viable alternative to lateral mass screws for posterior cervical fusion, especially in MISS contexts. Trajectory B, in particular, presents a feasible and safe alternative, reducing the risk of vertebral artery and spinal cord injury. Preoperative assessment and intraoperative technologies are essential for successful implementation. Biomechanical validation is needed before clinical application.

## Introduction

Lateral mass screw (LMS) fixation is considered as the standard of reference for posterior cervical fusion procedures between the spinal levels of C3 and C6 [12]. LMS fixation resembles and effective means of stabilization for the surgical treatment of degenerative, traumatic, inflammatory or neoplastic conditions of the cervical spine [30].

For LMS fixation, the most common and established screw trajectories are those according to Roy-Camille and Magerl. Roy Camille described a screw trajectory perpendicular to the center of the lateral mass and 10° medial to lateral angulation[34]. This technique was modified by Magerl et al. to an entry point 1 mm medial and 2 mm superior to the center of the lateral mass with a trajectory angulated 30° upwards accompanied with a 25° medial to lateral inclination [5]. Since the introduction of these techniques, multiple minor modifications have been described [14]. However, while not frequently occurring, severe complications, including injuries of the exiting nerve root [1, 23] or of the vertebral artery (VA), may occur [8, 9, 36, 38].

**Fig. 1:**
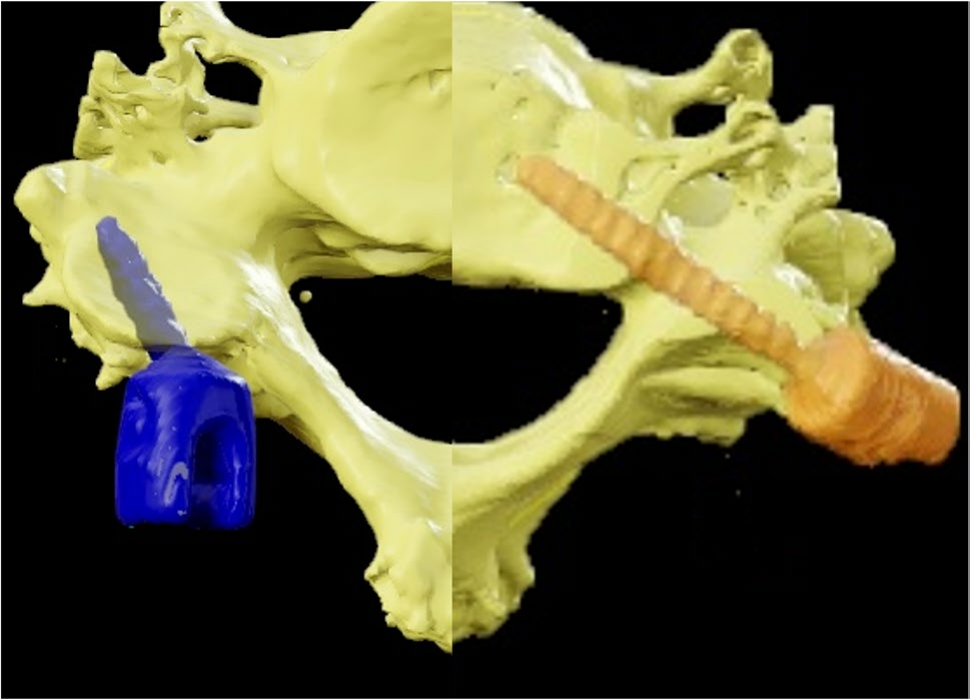
Lateralized vs. medialized screw trajectory in cervical spinal fusion surgery

The C2 and C7 vertebrae usually provide sufficient morphological dimensions for the insertion of cervical pedicle screws (CPS) [6, 16]. At end-levels of long cervical fusion constructs, CPS resembles the strongest osseous anchor points preventing mechanical failures. However, in combination with the use of LMS inserted in standard fashion, one disadvantage of CPS is the mismatch of entry sites in the axial plane. Pedicle screws are inserted in a medio-lateral trajectory compared to the LMS (Fig. 1). To achieve a harmonious connection of the implanted lateral mass to cervical pedicle screws, lateral off-set connectors, difficult three-dimensional rod bending or even omitting of adjacent LMS must be accepted to establish a rigid screw-rod alignment. The use of an in-line technique for matching LMS and CPS in multilevel constructs has been reported [30]. This technique enables easy in-line screw-rod connection and sagittal plane reconstructions for LMS within a long construct having CPS at end-levels [30]. Distinct situations, CPS even cannot be inserted safely as a result of unfavourable narrow or hypoplastic pedicle morphology. [26].

Over the past two decades minimally invasive spine surgery (MISS) techniques have gained increasing popularity. To date, only few attempts have been described regarding the performance of cervical instrumentation in a minimally invasive fashion [4, 7, 11, 17, 24, 27, 31, 32, 35]. However, the relatively medial entry point accompanied with the Magerl and Roy-Camille techniques close to the midline, where osseous and soft-tissue structures (spinous process, splenius and semispinalis muscles) hamper easy access to the entry site, resemble an anatomical restrained for establishment of standard MISS-approaches for instrumentation of the posterior cervical spine and cumber their usage in daily routine [17, 18].

**Fig. 2:**
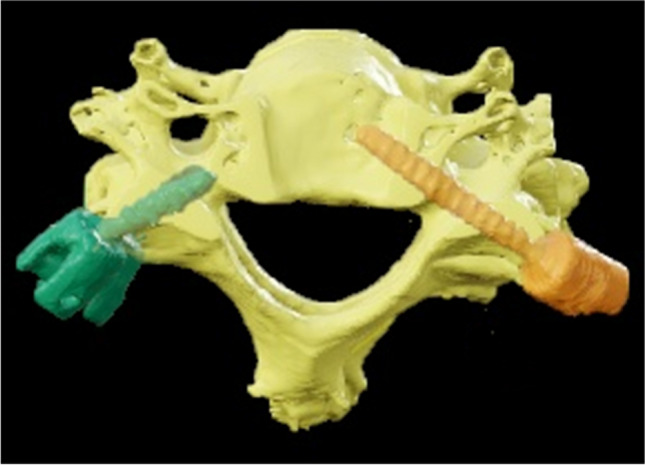
Schematic representation of cervical pedicle screw fixation (CPS) and pars interarticularis screw fixation (PIS)

To overcome some of the aforementioned limitations, the authors suggest a novel trajectory, which might be a valuable way mitigating LMS fixation as part of MISS-procedures. With the novel trajectory, screws are inserted at and through the pars interarticularis of the cervical vertebrae in a similar direction as with pedicle screws, but using approximately the same length and diameter as LMS fixation (Fig. 2). The herein called pars interarticularis screw (PIS) implanted in C3 to C6 show the potential to ease in-line instrumentation of both LMS and CPS and resembles an option as salvage strategy for failed LMS [10, 15].

The objective of the current study was to analyse two possible trajectories with PIS and to evaluate the feasibility of PIS for MIS techniques given its lateral entry point on the posterior cervical elements.

## Material and Methods

### Retrospective radiographic analysis.

The authors analysed reformatted CT scans of cervical spines performed from January to July 2018 at the authors’ institution. The data packages of 10 subsequent patients with gender 1:1 distribution were included in the study once they met the following conditions: Age between 18–80 years, absence of cervical trauma, infection, destruction, neoplastic or inflammatory disease, no prior history of cervical surgery, Caucasian origin. Measurements of the pars interarticularis morphology was done in 80 segments of 10 patients between C3 and C6 vertebral bodies. The pars interarticularis is the part of a vertebra located between the inferior and superior articular processes of the facet joint. When examined in the transverse plane, it lies between the lamina and pedicle. In the coronal plane, it is defined as the bony mass between the facets, anterior to the lamina and posterior to the pedicle. The pars interarticularis was divided in four quadrants to determine the ideal entry point.

All measurements were performed on both, the right and left side, using multiplanar reconstructions displayed in a DICOM viewer (Agfa Impax 6, Agfa-Health care, Mortsel, Belgium). With the use of multiplanar CT reconstructions, three-dimensional visualization was guaranteed and a reliable measurement of the two trajectories in all three planes was given.

### Analysis of two possible trajectories for PIS

**Fig. 3:**
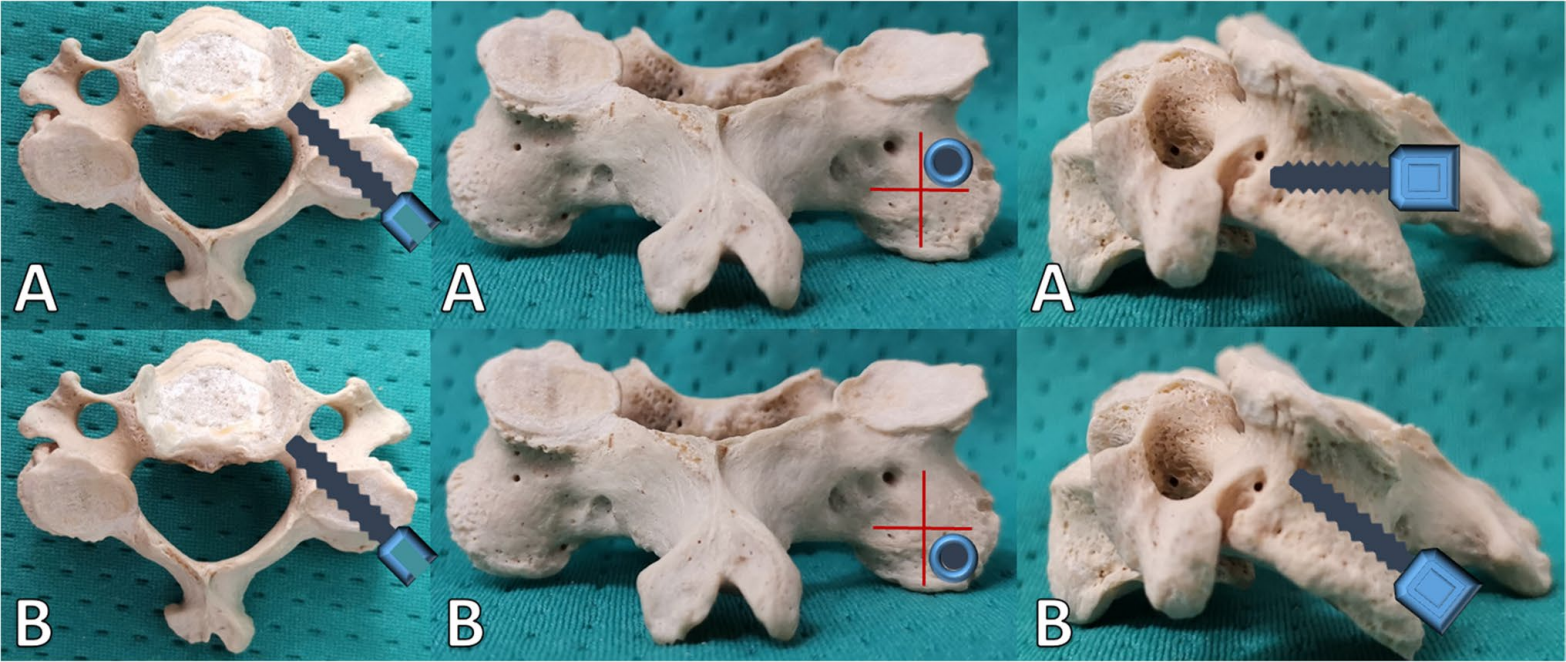
Entry point (outer upper quadrant of the lateral mass) and trajectory (medialized and caudalized) in model A (**a**) and model B (**b**)

Trajectory A was defined as an entry point in the upper outer quadrant of the lateral mass, approximately on the level of the upper edge of the pedicle. The trajectory was aimed in a medial and caudal direction towards the lower edge of the pedicle (Fig. 3a).

**Fig. 4:**
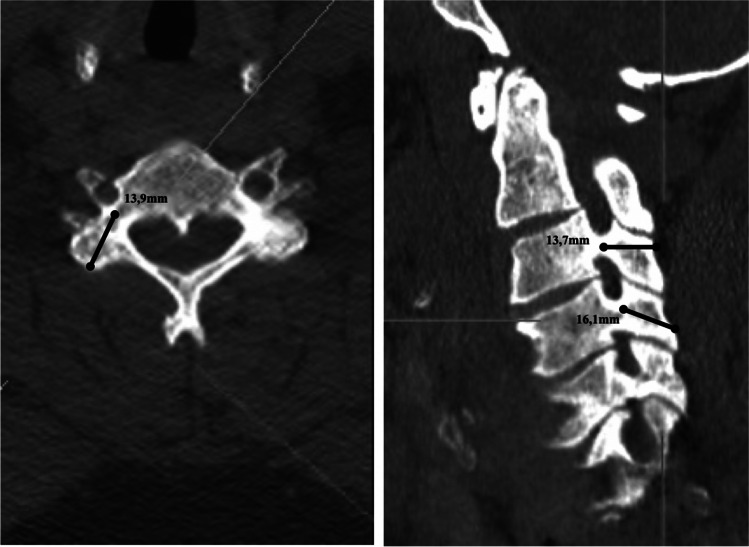
Example for sagittal and axial measurement in a multiplanar model

The entry point for trajectory B was determined in the lower outer quadrant approximately 5 mm cranial towards the lower edge of the lateral mass. The trajectory was aimed medially and cranially towards the upper edge of the pedicle (Fig. 3b). The authors defined the maximum possible screw length as the distance from the entry point to the upper and lower edge of the pedicle, respectively. Length measurements were performed in both the sagittal and axial slices and average values calculated for statistical use (Fig. 4).

As a control group and standard of reference, additional measurements of lateral mass screw (LMS) and pedicle screw (CPS) trajectory were performed in all patients, at all levels and on both sides.

**Fig. 5:**
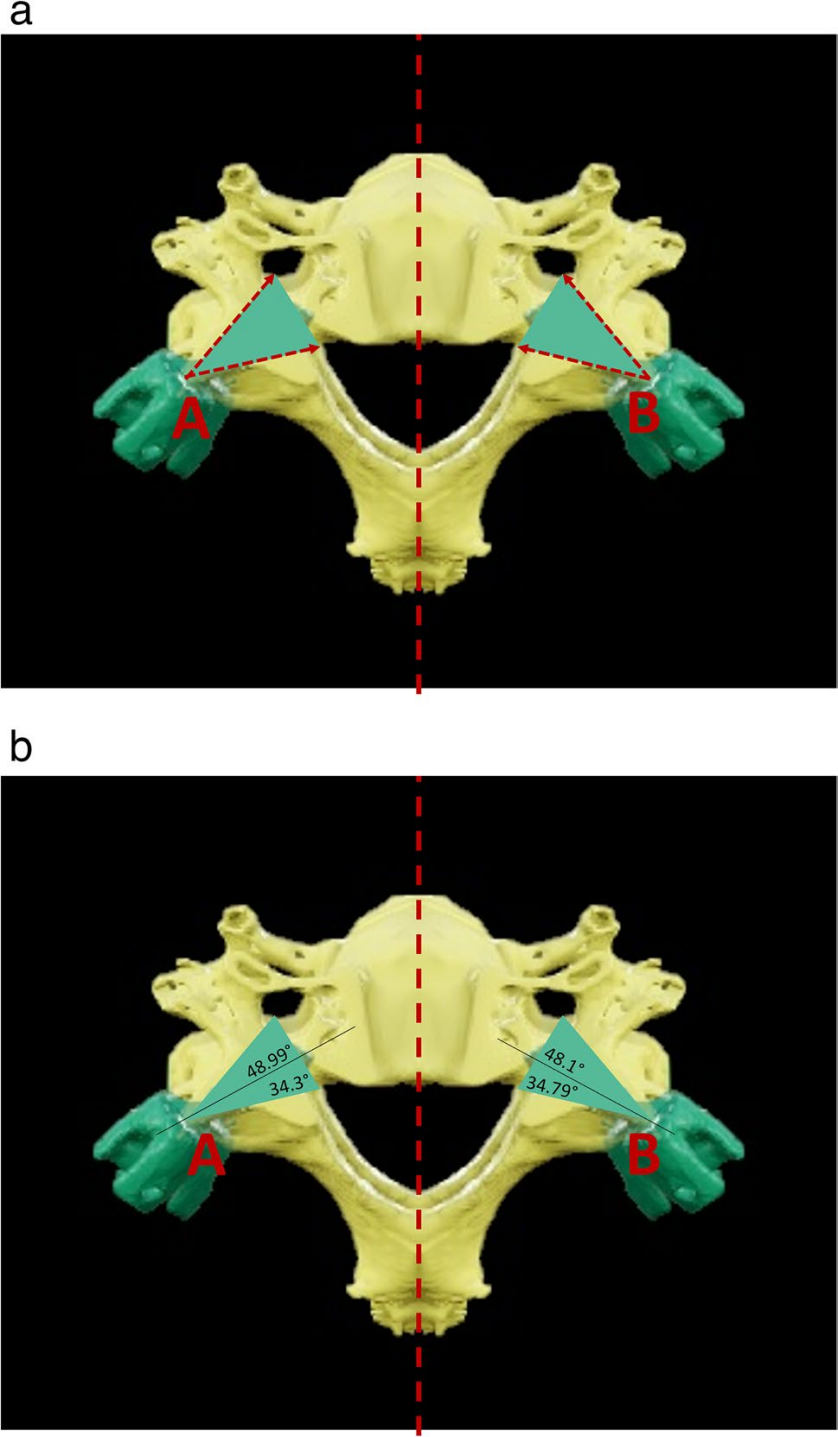
**a** Schematic measurement of angular deviations leading to penetration of the transverse foramen or the spinal canal. **b** Results of the angular deviations measurements without penetration of the transverse foramen (34°) or the spinal canal (48°)

A potential risk factor of the medial trajectory of the PIS involves penetrating the cortical shell of the pedicle compromising the spinal cord or the vertebral artery. For that reason, the authors measured the minimum distance from entry point A and entry point B (Fig. 5a) towards the spinal canal and the transverse foramen in the abovementioned multiplanar CT reconstruction model.

In order to standardize the measurements, a vertical line through the center of the spinal canal was drawn. We predefined entry points A and B in the ideal trajectory and measured the angular deviations that would lead to penetration of the transverse foramen and the spinal canal, respectively, as shown in Fig. 5a.

Demographic information was collected, including patients’ age and sex. All measurements were performed by two neurosurgical residents and by an attending neurosurgeon to evaluate the “interobserver” reliability.

### Statistics

The results of the measurements were averaged for each measured level between C3 and C6 and statistically analysed. All values are expressed as mean ± standard deviation (SD). The Kolmogorov–Smirnov test was used for testing normal distribution. Frequencies were compared by the chi- square and Fisher’s exact tests. The level of significance was set to p < 0.05. All statistical evaluations were performed with SPSS Version 21.0 (IBM Corp. Released 2012. IBM SPSS Statistics for Mac OS X, Version 21.0, NY: IBM Corp.). Figures were designed using Adobe Photoshop Lightroom CC 2020 (Version 2.4.1 for Mac OS X, Adobe Inc., San José, USA).

## Results

The mean age of the patients was 62 ± 11 years (range 43–76 years), and the gender distribution of the measured CT scans was 1:1 between men and women. There was no statistical difference in any measurement between the right and left sides or between male and female patients.

Significantly longer pars lengths for screw placement were observed with entry point B (15.69 ± 0.65) compared to entry point A (12.51 ± 0.24; p < 0.01). 90% of measurements using trajectory A were larger than 12 mm, whereas 95% of measurements with trajectory B where larger than > 14 mm when using trajectory B. Regarding comparison of Trajectory A and B, there were no significant differences between maximum possible angulation lateral towards the transverse foramen (A: 34.3 ± 2.36° vs. B: 34.79° ± 1.94°; p > 0.05) or medial towards the spinal canal (A: 48.99° ± 1.95° vs. 48.1° ± 2.23°; p > 0.05; Fig. 5b).

The mean length of lateral mass screws did not differ significantly from the length of pars interarticularis in entry point B (16.99 mm ± 0.36 mm; p > 0.05) whereas the length of pedicles was significantly longer (30.70 mm ± 0.92 mm).

## Discussion

**Fig. 6:**
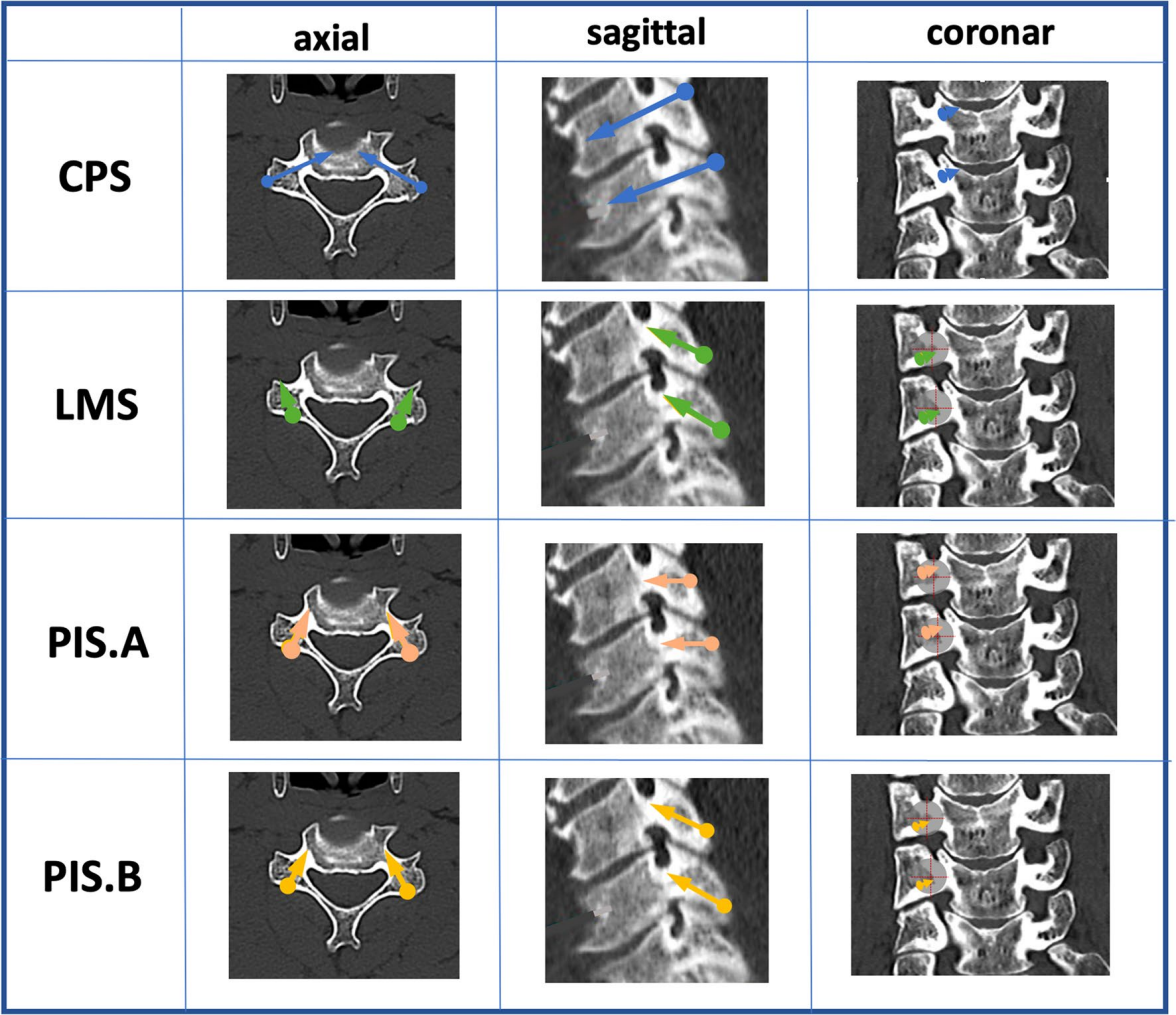
Schematic overview of the various trajectories. CPS: pedicle screw fixation; LMS: lateral mass screw fixation; PIS-A: pars interarticularis screw fixation, trajectory A; PIS-B: pars interarticularis screw fixation, trajectory B

Various techniques are available for screw fixation of the cervical spine. (Fig. 6) LMS fixation is considered as the gold standard for posterior cervical fusion procedures between the C3 and C6 vertebral body. Although not frequently reported, serious complications may occur utilizing these trajectories. Most common complications involve the injury of the exiting nerve root [1, 23] or injuries of the vertebral artery [8, 9, 36, 38]. PIS are already used in C2 fixations [21] and are known to have a minimized risk of VA injury due to the lateral to medial angulation. The aim of the presented analysis was to radiologically investigate two pars interarticularis trajectory of the subaxial spine with the advantages of a minimized risk of injury of adjacent structures and the potential utilization of this novel screw trajectory in minimally invasive procedures.

Due to recent technological advances, such as the implementation of computer assisted navigation, most spine procedures can now be performed safely in a minimally invasive fashion in the lumbar, thoracic and more recently also in the cervical spine. However, to date, few reports of MIS posterior cervical fusion have been published [2, 3, 36, 37]. In all reported case series, lateral mass screws have been used. Most notably was the case series by Wang et al., reporting about long-term outcomes of 18 patients who received minimally invasive lateral mass screw fixation [35]. While reporting good clinical outcomes, the authors used 18-22 mm tubular retractors indicating that this MIS technique still requires significant exposure in order to safely use a lateral mass trajectory. According to the proposed technique of Magerl et al. and to achieve a sufficient mediolateral screw trajectory for an optimal LMS placement within the lateral mass, a resection of the spinous process might occasionally be necessary. In case of a minimally invasive approach, however, the resection of the spinous process is not possible. As a result, PIS might represent a reliable alternative, since our radiological evaluation did not show significant differences in screw length comparing LMS to PIS using the proposed trajectory B, whereas trajectory A showed significantly shorter screw lengths in comparison to LMS. The measured entry points of the present study represent a lateral to medial trajectory. As a result, less exposure will be necessary for safe screw placement without the need of resecting supra- or infraspinous ligaments or the spinous process. Moreover, easier rod adjustment, especially in case of extending the posterior stabilization to a pedicle screw routinely used in the vertebral body C7, could be performed.

Navigation in cervical fusion remains a point of discussion, not only due to various positions of the navigation array (Mayfield clamp vs. spinous process). Nevertheless, accuracy seems to be comparable in both options either positioning the reference array on the Mayfield clamp or the spinous process, so that the reference array might be positioned at the Mayfield clamp in the proposed PIS option in order to assume accuracy [32].

Our results show that both described entry points represent safe and feasible alternatives for standard LMS placement for posterior cervical fusion. Using entry point A, 90% of the pars interarticulares measured were at least 12 mm in length. In entry point B, the pars length was ≥ 14 mm in 95% of the measured vertebrae. Therefore, the utilization of standard lateral mass screws appears to be feasible in the majority of patients. Furthermore, the range of trajectories within the safe zone between the entry point, the spinal cord and the vertebral artery is relatively wide, ranging from 35° to 48° for both entry points. Consequently, the risk to injure delicate structures should be significantly reduced. This is particularly true because medial angulation required in PIS is reduced to a minimum of 10–15% [22]. Therefore, this novel trajectory is also more applicable performing for MISS, as the application of tubular approaches is more practicable for lateral to medial trajectories without the space occupying effect of medial structures compared to the “gold standard” LMS. Amongst others, this angulation presents a common limitation for MISS LMS, especially in C7, where the required lateral angulation is often hardly achieved [27].

**Fig. 7:**
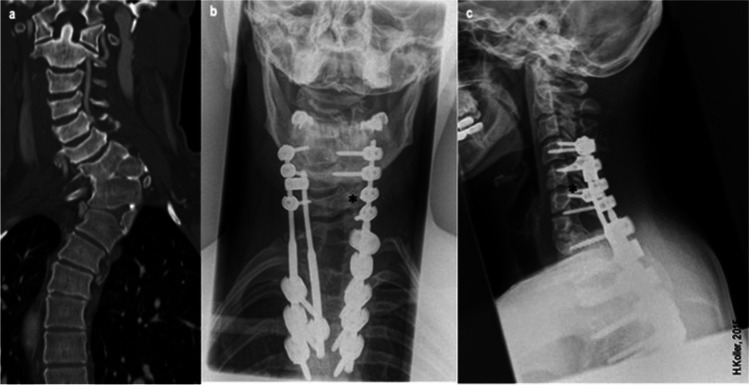
Congenital cervical scoliosis in female 28 years old patient. Treatment with hemivertebra resection C7-T1 & T1-2. Decompression of roots C5-T1, pedicle screws C4 + C5, T1, and T2-4, At level of dysplastic vertebral anatomy a short CPS at C6 was placed right side (*)

Additionally, the outlined method may have notable advantages in circumferential procedure (e.g. anterior cervical corpectomy and fusion procedures) with the need of an additional dorsal instrumentation (Fig. 7), as the possibility of MIS screw-implantation may lower loss of blood and length of operation time accompanied with all other advantages of MISS in these two-staged procedures. A supplemental posterior approach after 1-, 2- or multilevel corpectomy procedures preserves primary construct stability [19, 29, 37]. Biomechanical studies show that a significantly ROM reduction can be obtained, so that implant-related complications in a real clinical scenario might be prevented. Nevertheless, a necessary second posterior approach might lead to a non-negligible increase in the rate of perioperative complications [13, 20, 25, 28, 33]. As a consequence, the second posterior approach might be performed in the same session with lower loss of blood, length of operation time and tissue damage due to PIS placement through a MISS approach. The literature provides evidence, that two-timed procedures increase morbidity and mortality as described by Boakye et al., which could therefore be prevented with this novel screw trajectory [28].

While our findings underscored the promising anatomical feasibility and possible biomechanical advantages of Trajectory B, it is essential to consider the role and potential of Trajectory A and maybe also its comparison with a short pedicle screw. Trajectory A, with its upper outer quadrant entry and horizontal trajectory, demonstrated comparable safety margins and potential in initial evaluations. However, our focus on Trajectory B in this study was driven by its longer length and therefore better comparability with LMS, which facilitates robust screw fixation and potentially reduces the risks, as well as the potentially reduced stability associated with traditional trajectories. Future studies could further clarify the comparative benefits of Trajectory A versus short pedicle screws, particularly in terms of biomechanical stability and clinical outcomes. Still, the zone of interest in terms of screw purchase might be the cortical transition zone between pedicle and vertebral body which may not be reached sufficiently by a so-called short pedicle screw. Maybe this future comprehensive evaluation would provide clinicians with a more nuanced understanding of optimal screw trajectories for posterior cervical fusion procedures.

### Limitations

The described study was solely an imaging study and may not reflect the intraoperative anatomical conditions appropriately. Additionally, the authors performed 80 measurements in ten patients, consequently individual anatomical abnormalities and variations may not be considered. Prior translation to clinical application, biomechanical studies are mandatory in order to drive final conclusions of feasibility and safety of these new trajectories.

## Conclusion

Pars interarticularis screws are an alternative method for posterior fusion of the cervical spine with less risk of vertebral artery and spinal cord injury according to our measurements. Anatomical evaluations using CT scans suggests that 95% of partes interarticulares present with at least 14 mm when using the novel trajectory B. If trajectory A is used, one should note that 10% of partes may have a length shorter than 12 mm. Consequently, considering the importance of neurovascular structures in this region, a careful preoperative evaluation of patient imaging is necessary to determine the appropriate screw insertion technique and, especially, the implant length. Supportive measurements, such as intraoperative navigation, may be necessary for patients with difficult anatomy.

## Data Availability

Not applicable.
